# The changing relationship between health risk behaviors and depression among birth cohorts of Canadians 65+, 1994–2014

**DOI:** 10.3389/fpsyt.2022.1078161

**Published:** 2022-12-21

**Authors:** Guang Yang, Carl D’Arcy

**Affiliations:** ^1^School of Public Health, University of Saskatchewan, Saskatoon, SK, Canada; ^2^Department of Psychiatry, College of Medicine, University of Saskatchewan, Saskatoon, SK, Canada

**Keywords:** population 65+, depression, physical activity, smoking, alcohol use, historical trends, birth cohorts, period effects

## Abstract

**Background and objective:**

The older adult residents of Canada form an increasingly larger proportion of the population and are becoming better educated and have more income. Depression is a common mental disorder, particularly among seniors. Several health risk behaviors–physical inactivity, tobacco use, and alcohol consumption–are linked to mental health problems. This study examines whether these health risk behaviors and their association with depression among Canadians 65+ born in eight cohorts between 1910–1914 and 1945–1949, have changed.

**Methods:**

Pooled data drawn from 11 nationally representative health surveys conducted by Statistics Canada between 1994 and 2014 are analyzed–88,675 survey participants met inclusion criteria. Depression was assessed by the Composite International Diagnostic Interview–Short Form. Health risk behaviors examined were *physical activity/inactivity*, *smoking*, and *alcohol use*. A Cochran Armitage trend test for categorical outcomes and a log-binomial modeling for binary outcomes were used to estimate the risk ratios across cohorts.

**Results:**

The proportions of Canadians 65+ who are physically active, regular drinkers, and regular smokers have increased; however, depression prevalence fluctuated non-significantly. Depression increased among all health risk behaviors, particularly in recent birth cohorts. Depression among physically inactive seniors, current smokers, and non-drinkers was significantly higher than among active, non-smokers, and regular drinkers (all *P* < 0.05). Physical inactivity and smoking-attributable depression risk showed an increasing linear trend across birth cohorts (RR = 1.67, *P* < 0.001; RR = 1.79, *P* < 0.001). For seniors born between 1915 and 1944, regular drinking was associated with a significant decrease in depression (all *P* < 0.001), but the protective effects of regular drinking became non-existent in the most recent 1945–1949 birth cohort (RR = 1.09, *P* < 0.05, after adjusting for covariates).

**Conclusion:**

Inactivity and smoking were consistently associated with a significantly increased risk of depression among Canadian residents 65+, with smoking becoming more firmly connected to depression risk in more recent birth cohorts. In contrast, moderate alcohol use was associated with a decreased risk of depression, but that protective effect ceased in most recent birth cohort. Identifying the changing relationships between health risk behaviors and depression is meaningful for developing prevention strategies for depression and other emotional and mental health problems.

## Introduction

Promoting healthy lifestyle behaviors is considered an effective strategy for preventing and/or treating diseases (1). The secular trend in healthy lifestyles throughout the 20th and 21st centuries shows that a growing number of people have become aware of the role of lifestyle changes in disease prevention. Maintaining healthy lifestyles, such as a healthy diet, a healthy body weight, and being physically active, has a large impact on morbidity and mortality. A study of the USA population shows that a healthy lifestyle can significantly lower premature mortality and prolong life expectancy (2). In a variety of countries, perhaps as a result of to research and social messaging, there are beneficial changes in lifestyle occurring at the population level. Even in their later years, older individuals show they are capable of learning new skills and participating in social and physical activities.

Governments and public health officials worldwide have promulgated and promoted guidance on healthy lifestyle factors in the pursuit of improving physical and mental health. It is well acknowledged that physical activity is crucial for maintaining independence, preventing disease, and enhancing the quality of life, particularly among older individuals. In order to embrace health benefits, such as a decreased risk of mortality, cardiovascular disease, depression, and dementia in older adults, guides such as the 24-h Movement Guidelines (3), which incorporate physical activity, sedentary behavior, and sleep, have been promoted in Canada. However, physical inactivity among older adults is still a significant risk factor for increasing the burden of disease, especially globally (4).

Also, older adult smokers are more likely than non-smokers to experience worse outcomes from major age-related disorders and have poorer management of those disorders (5). Smoking may also reduce the effectiveness of vaccines and raise the risk of many cancers in the elderly population (6). Depressed people have been found to be more likely to smoke, which may be due to nicotine’s powerful neurophysiologic effect (7, 8). A Canadian longitudinal study using national survey data showed that prior smoking was a significant risk factor for incident depression (9). Additionally, there is a great deal of evidence demonstrating complex behavioral and neurophysiologic interactions between smoking and mental health (10). Research supporting a causal relationship between smoking and depression shows a reduction in depression among those who quit smoking (11). Even in later years, quitting smoking can considerably lower mortality and enhance the quality of life, particularly in older people with underlying conditions associated with smoking, e.g., COPD (12). However, an analysis of a repeated multi-wave cross-sectional household survey in England, found that despite being less dependent on nicotine than younger smokers, older smokers appeared less motivated to quit smoking and were less likely to be offered support to quit smoking (13).

According to an analysis of the National Health Interview Surveys (USA) from 1997 to 2014, the number of Americans aged 60 and older who drink has steadily increased, especially among women (14). Some special considerations facing older adults who drink alcohol include a higher sensitivity to alcohol, increased health problems, and harmful interactions with medications. As people age, their bodies process alcohol more slowly, making them more vulnerable to the negative effects of alcohol. Families, friends, and healthcare professionals may fail to take elderly people’s drinking into account. This may be the case because older persons’ drinking-related side effects are sometimes misinterpreted as aging-related problems, such as balance issues.

Depression, one of the most prevalent mental illnesses in older adults, affects approximately 7% of the senior population worldwide (15). By 2020, depression has become the second leading cause of death and is thought to have a secondary role in 50–70% of suicides among the elderly (16). Healthy lifestyles have been identified to contribute to a lower risk of chronic diseases and mental disorders, especially depression (17–19). Even though the majority of cases of depression cannot be prevented, healthy lifestyle changes can have benefits for older individuals’ mental health in the long run. Previous studies have demonstrated that leading a healthy lifestyle, which includes regular exercise, physical examinations, and following dietary rules, can reduce the risk of depression in older people (20). It has been shown in many populations that those who do not live a healthy lifestyle are more prone to experiencing depression (21–23). For instance, there is strong evidence for an inverse association between physical exercise and depression in the general population (24) and older individuals (25). Physical activity has been identified as a favorable non-pharmaceutical strategy for treating and preventing depressive symptoms in older adults (26). Bidirectional associations between tobacco use, heavy drinking, and depression among older adults have been widely observed (27). Findings from prospective, longitudinal studies have validated that smoking and alcohol drinking have a long-term impact on later depression and vice versa (28, 29).

Evidence shows that individual behaviors and mental health have increasingly changed at the population level (30). People born in certain birth cohorts come of age at specific periods of history and experience disparate socio-economic and political environments. As the imminent sociologist C Wright Mills (31) wrote, “Neither the life of an individual nor the history of a society can be understood with understanding both.” Each birth cohort lives and ages within a historical capsule. These unique economic characteristics, political conditions, and social norms vary from time to time in different countries. They become essential exposures in the development of lifestyle behaviors and psychological outcomes. Identifying population-level factors influencing unhealthy lifestyles and psychological consequences have significant public health implications.

How do the associations between unhealthy lifestyle factors and depression vary across and among individuals born in different periods as they age? We examine those questions among Canadians aged 65+ born between the 1910s and 1950s. It is an unexplored topic. This study aims to provide a comprehensive picture of how unhealthy lifestyle factors, the prevalence of depression, and their associations change among Canadians 65+ across the eight birth cohorts born between 1910 and 1949, thus having been exposed to diverse epoch experiences as they have aged.

## Materials and methods

### Data source and study population

The current study was based on the pooled data on Canadian adults 65+ drawn from the public use microdata files (PUMF) of the National Population Health Survey (NPHS) series (32) and the general health cycles of the Canadian Community Health Survey (CCHS) series (33), all nationally representative omnibus health surveys. These large interview surveys were conducted in either of Canada’s official languages, French or English, by Statistics Canada, the country’s national statistical agency. The NPHS began in 1994 and was conducted every 2 years (in 1994/1995, 1996/1997, and 1998/1999). It has both nationally representative cross-sectional and longitudinal components. Here we examine the cross-sectional data. The CCHS, a successor to the NPHS, is a repeated, cross-sectional, nationally representative omnibus health survey of Canadians ages 12+ years in the ten provinces and three territories of Canada conducted by Statistics Canada and Health Canada. A detailed description of the study design and methodological information on the CCHS is available elsewhere (33). Since Canada is a confederation of provinces and territories and health is constitutionally a provincial responsibility, not all items asked in the annual health surveys are necessarily asked in all provinces or territories every year. Consequently, the CCHS contains both core contents asked in all provinces and territories and optional content modules that provinces and territories can opt into or out of in any collection year. The epidemiological measurement of depression was optional in the CCHS general surveys. So, provinces or territories can opt in or out of collecting data on depression; thus, sample sizes, while considerable, can vary in any given survey year. In brief, the NPHS and CCHS collect a standard set of information related to demographic and socio-economic characteristics, health status, healthcare utilization, and health determinants for the Canadian population. Data from eleven surveys from 1994 to 2014 was used in our current study. This study examined data from survey participants over 65 years of age with complete data on depression and health risk behavior modules. Supplementary Table 1 details the data collected on each survey/year and the survey sample sizes for the respondents 65+ sample. The current study has a total sample of 88,675 participants 65+: the NPHS 1994/1995 contributed 2,792 participants; NPHS 1996/1997–8,877; NPHS 1998/1999–2,436; CCHS 2001–18,358; CCHS 2003–7,259; CCHS 2005–10,817; CCHS 2007/2008–7,331; CCHS 2009/2010–9,959; CCHS 2011/2012–5,415; CCHS 2013/2014–10,455 participants; CCHS 2014–5,406.

Ethical approval for data collection was provided by Statistic Canada processes. All survey participants provided written consent to be surveyed. This current study involves the secondary analysis of publicly available anonymized survey data. There are no individual identifying information in the data. No institutional ethical clearance was required for this specific study. We assert that all procedures contributing to this work comply with the ethical standards of the relevant national and institutional committees on human experimentation and with the Helsinki Declaration as revised in 1989.

### Measures

#### Dependent variable

*Major depressive episode* in the past 12 months, as assessed using the Composite International Diagnostic Interview–Short Form (CIDI-SF) (34). The CIDI-SF is a structured diagnostic instrument aiming to diagnose diseases according to the Diagnostic and Statistical Manual of Mental Disorders-III (DSM-III) and International Classification of Disease-10 (ICD-10) criteria. The measure indicates the probability that a respondent would be diagnosed as having experienced a major depressive episode in the past 12 months. A CIDI-SF score of 5 or greater, which indicates a high probability of depression, was categorized here as a depression case.

#### Independent variables

##### Birth cohort

Based on the birth year, all survey participants were classified into eight birth cohorts (1910–1914, 1915–1919, 1920–1924, 1925–1929, 1930–1934, 1935–1939, 1940–1944, 1945–1949) calculated by using the survey year minus their age.

##### Health risk behaviors

Three measures of health-related behaviors were included in the study: physical activity index, smoking status, and type of drinker.

The *physical activity index*, a survey-derived variable, was based on responses to questions about the frequency, nature, and duration of participation in leisure-time physical activity over the past 3 months. Average energy expenditure was calculated by multiplying the number of times they engaged in each activity, the average duration of participation in hours, and the metabolic equivalent of task value assigned to each activity (expressed in kilocalories expended per kilogram of body weight per hour of activity). Participants were categorized as: *active* [≥3.0 kcal/kg/day (KKD)], *moderate active* (1.5–2.9 KKD), and *inactive* (<1.5 KKD) (33, 35).

*Alcohol consumption* was also a survey-derived variable based on respondents’ answers to a series of questions concerning alcohol use. Respondents were categorized as: regular drinker (drank alcohol ≥ one time per month), occasional drinker (drank alcohol < one time per month), and non-drinker (did not drink in the past 12 months).

*Smoking status* was measured by asking participants a series of questions concerning current and past cigarette smoking habits. Survey respondents were labeled as: daily smoker, occasional smoker but former daily smoker, always an occasional smoker, former daily smoker, former occasional smoker, or never smoked. We recoded those categories into three categories for our analysis: current smoker (daily smoker, occasional smoker but former daily smoker and always occasional smoker), former smoker (former daily smoker and former occasional smoker), and non-smoker (those who never smoked).

##### Sociodemographic characteristics

We controlled for the following variables in all adjusted regression models: *sex* (male/female), *educational attainment* (less than secondary/secondary graduation/some post-secondary/post-secondary graduation), *body max index* [BMI, calculated as body weight in kilograms divided by squared height in meters, kg/m^2^; underweight (BMI < 18.5), normal weight (18.5 ≤ BMI < 25), overweight (25 ≤ BMI < 30), and obese (BMI ≥ 30)], *household income quintile* (lowest/lower middle/middle/upper middle/highest), *marital status* (married/common-law, widowed/separated/divorced, and single), *immigration status* (Yes/No), and *language use* (English only/French only/English and French/Other).

### Statistical methods

The NPHS and CCHS surveys used a multistage stratified sampling design. Each survey participant was assigned a sampling weight, and all the percentages and rates were weighted to correct for oversampling and represent the national community-dwelling population.

A Cochran Armitage (CA) trend test for categorical outcomes was used to analyze how the proportion of different lifestyles and the rate of depression change across the birth cohorts. A positive *Z*-value indicates increasing linear trends from the earliest to the most recent birth cohorts, and a negative value shows a decreasing trend.

A Bayesian approach to solve the convergence problem of a log-binomial model in binary outcome was built to estimate risk ratio (RR) to analyze the changes in percentage and rate across birth cohorts (36).

A binomial generalized estimating equation model with an identity link was used to estimate the risk difference of depression for different lifestyles (37).

Differences in the associations between health behavioral risk factors and depression across birth cohorts were tested by fitting a log-binomial regression model with a linear cohort term, a three-level behavior term, and a behavior-by-cohort interaction term. The coefficients of interaction terms were used to test whether the relationships between behavioral risk factors and cohort varied by cohort, with positive coefficients reflecting increasing and negative estimates manifesting decreasing associations between behavioral risk factors and depression across cohorts.

All of the above log-binomial regression models were adjusted by adding sex, educational attainment, BMI, household income, marital status, immigration status (Yes/No), and language use. Meanwhile, all these analyses were repeated using the survey year instead of the birth cohort to delineate changes across time periods.

All analyses were conducted in R (4.0.3). Both OpenBugs 3.2.3 and R BRugs packages were used to carry out the Bayesian approach for the log-binomial model using Markov Chain Monte Carlo (MCMC) (38).

## Results

### Prevalence and trend in health risk behaviors across birth cohorts

The characteristics of all participants are shown in Table 1. The majority of participants were females, of normal weight, the middle level of household income, non-immigrants, and English-only speakers in most cohorts. The earlier-born cohorts are less educated and more likely to be widowed/separated/divorced compared to the later-born cohorts who are better educated and married.

**TABLE 1:** Characteristics of the Canadian residents 65+ across the eight birth cohorts from 1910s to 1940s.

Variables	1910–1914 (542) *n* (%)^α^	1915–1919 (8,703) *n* (%)^α^	1920–1924 (11,920) *n* (%)^α^	1925–1929 (18,075) *n* (%)^α^	1930–1934 (18,586) *n* (%)^α^	1935–1939 (13,955) *n* (%)^α^	1940–1944 (11,302) *n* (%)^α^	1945–1949 (5,592) *n* (%)^α^
**Sex**								
Males	174 (35.54)	2,924 (40.25)	4,473 (40.97)	7,358 (44.57)	7,815 (46.43)	6,161 (46.88)	5,153 (49.75)	2,483 (46.47)
Females	368 (64.46)	5,779 (59.75)	7,447 (59.03)	10,717 (55.43)	10,771 (53.57)	7,794 (53.12)	6,149 (50.25)	3,109 (53.53)
**Education**								
Less than secondary	340 (56.98)	5,066 (55.26)	6,277 (51.65)	8,729 (46.25)	8,186 (40.92)	4,886 (31.21)	3,078 (24.09)	1,588 (24.45)
Secondary graduation	50 (11.93)	1,088 (12.91)	1,608 (14.75)	2,443 (14.12)	2,587 (14.80)	1,892 (13.44)	1,572 (14.54)	994 (18.02)
Other post-secondary	101 (22.16)	713 (13.54)	914 (12.87)	1,298 (13.18)	901 (8.50)	592 (4.60)	502 (4.27)	186 (3.50)
Post-secondary graduation	51 (8.93)	1,836 (18.29)	3,121 (20.73)	5,605 (26.45)	6,912 (35.78)	6,585 (50.75)	6,150 (57.10)	2,824 (54.03)
**BMI (Kg/m^2^)**								
Underweight	19 (3.10)	387 (3.42)	363 (2.05)	469 (1.85)	412 (2.08)	262 (2.12)	165 (1.44)	64 (1.12)
Normal weight	342 (64.08)	4,505 (54.43)	5,546 (46.39)	7,773 (42.94)	7,651 (41.61)	5,208 (39.22)	3,979 (36.60)	1,812 (34.18)
Overweight	138 (26.57)	2,885 (32.71)	4,430 (38.26)	7,093 (40.20)	7,292 (38.98)	5,711 (40.59)	4,528 (40.90)	2,256 (40.97)
Obesity	43 (6.25)	926 (9.45)	1,581 (13.30)	2,740 (15.01)	3,231 (17.33)	2,774 (18.07)	2,630 (21.06)	1,460 (23.73)
**Household income**								
Lowest	96 (12.57)	551 (5.06)	1,060 (5.56)	2,528 (6.97)	2,925 (9.22)	2,421 (12.33)	2,101 (13.14)	906 (11.86)
Lower middle	185 (26.08)	2,678 (24.25)	3,194 (20.26)	5,063 (19.69)	5,708 (24.31)	4,922 (32.76)	4,195 (35.08)	1,802 (32.13)
Middle	190 (36.53)	3,627 (42.81)	4,540 (41.36)	5,731 (37.53)	5,058 (31.09)	3,367 (25.38)	2,436 (23.03)	1,328 (24.36)
Upper middle	62 (21.13)	1,534 (22.18)	2,480 (25.82)	3,566 (26.85)	3,449 (23.65)	2,021 (17.14)	1,265 (13.56)	716 (14.22)
Highest	9 (3.71)	313 (5.70)	646 (7.00)	1,187 (8.96)	1,446 (11.73)	1,224 (12.40)	1,305 (15.19)	840 (17.43)
**Marital status**								
Married/Common-law	136 (35.32)	2,599 (45.44)	4,674 (53.99)	8,181 (62.73)	9,044 (63.28)	7,847 (67.21)	6,612 (68.68)	3,414 (68.65)
Widowed/separated/divorced	363 (56.97)	5,602 (49.04)	6,597 (41.85)	8,790 (32.09)	8,373 (31.52)	5,229 (27.58)	3,912 (25.90)	1,700 (23.68)
Single	43 (7.71)	502 (5.52)	649 (4.16)	1,104 (5.18)	1,169 (5.20)	879 (5.21)	778 (5.42)	478 (7.67)
**Immigration status**								
Yes	129 (29.21)	1,519 (20.45)	2,361 (23.79)	3,061 (24.10)	2,604 (21.56)	1,757 (18.93)	1,044 (13.25)	389 (12.32)
No	413 (70.79)	7,184 (79.55)	9,559 (76.21)	15,014 (75.90)	15,982 (78.44)	12,198 (81.07)	10,258 (86.75)	5,203 (87.68)
**Language use**								
English only	369 (63.50)	6,487 (63.13)	8,563 (60.65)	12,250 (57.72)	11,864 (57.04)	8,329 (51.04)	6,036 (59.96)	2,735 (54.26)
French only	37 (10.87)	591 (14.54)	1,149 (13.46)	2,843 (17.85)	4,512 (25.84)	4,018 (34.48)	4,442 (29.07)	2,793 (43.55)
English and French	38 (8.10)	794 (12.55)	1,381 (15.89)	1,980 (15.31)	1,968 (14.96)	1,424 (11.66)	711 (8.42)	55 (1.83)
Other	100 (17.53)	831 (9.78)	827 (10.00)	1,002 (9.32)	242 (2.16)	184 (2.82)	113 (2.55)	9 (0.35)

^α^Numbers are unweighted, percentages are weighted.

**FIGURE 1:**
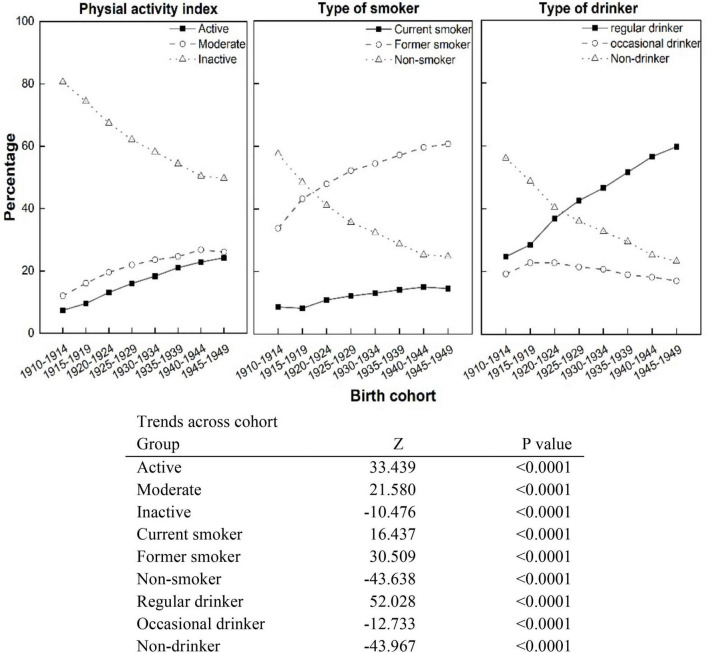
Trends in physical activity index, smoking status, and type of drinker across birth cohorts, from the 1910s to 1940s, Canadian residents 65+.

Our first analysis concerns the frequency and trends of health risk factors across birth cohorts. Table 2 and Figure 1 show the frequency and trends in physical activity index, smoking status, and type of drinker across birth cohorts. For the *physical activity index*, the proportion of “active” and “moderately active” Canadians 65+ increased from the 1910s to 1940s birth cohorts. For *cigarette smoking*, there was a significant positive linear trend observed in the percentage of “current smokers” with an average increase of approximately 8.8% (RR = 1.09, *P* < 0.0001) per cohort, as we move from the 1910s to 1940s birth cohort. As we can see from the data on *alcohol use* in Figure 1, there is an increase in the proportion of regular drinkers among those 65+ from 24.72% in the 1910–1914 birth cohort to 59.71% in the 1945–1949 birth cohort; this has been accompanied by a significant decrease in the proportion of “occasional drinkers” and “non-drinkers,” a linear trend, all *P*-values < 0.0001.

**TABLE 2:** Frequency of physical activity, smoking status, and type of drinker across birth cohorts for Canadian residents 65+.

Birth cohort	Physical activity index			Smoking status			Type of drinker		
			
	Active % (95% CI)	Moderate % (95% CI)	Inactive % (95% CI)	Current smoker % (95% CI)	Former smoker % (95% CI)	Non-smoker % (95% CI)	Regular drinker % (95% CI)	Occasional drinker % (95% CI)	Non-drinker % (95% CI)
1910–1914 (*N* = 542)	7.38 (5.17, 9.59)	11.99 (9.25, 14.74)	80.63 (77.29, 83.97)	8.67 (6.29, 11.05)	33.76 (29.77, 37.76)	57.57 (53.39, 61.74)	24.72 (21.08, 28.37)	19.19 (15.86, 22.51)	56.09 (51.90, 60.28)
1915–1919 (*N* = 8,703)	9.54 (8.92, 10.15)	16.07 (15.30, 16.85)	74.39 (73.47, 75.31)	8.25 (7.67, 8.83)	43.21 (42.17, 44.26)	48.54 (47.48, 49.59)	28.51 (27.56, 29.46)	22.76 (21.88, 23.64)	48.73 (47.68, 49.78)
1920–1924 (*N* = 11,920)	13.06 (12.46, 13.67)	19.53 (18.82, 20.24)	67.41 (66.57, 68.25)	10.95 (10.39, 11.51)	47.92 (47.02, 48.82)	41.13 (40.25, 42.02)	36.88 (36.01, 37.75)	22.76 (22.01, 23.51)	40.36 (39.48, 41.24)
1925–1929 (*N* = 18,075)	15.96 (15.42, 16.49)	21.89 (21.29, 22.50)	62.15 (61.45, 62.86)	12.18 (11.70, 12.65)	52.17 (51.42, 52.87)	35.65 (34.95, 36.35)	42.62 (41.90, 43.34)	21.41 (20.81, 22.01)	35.97 (35.27, 36.67)
1930–1934 (*N* = 18,586)	18.28 (17.72, 18.83)	23.53 (22.92, 24.14)	58.19 (57.48, 58.90)	13.10 (12.61, 13.58)	54.44 (53.72, 55.15)	32.46 (31.79, 33.14)	46.59 (45.87, 47.31)	20.69 (20.11, 21.28)	32.72 (32.04, 33.40)
1935–1939 (*N* = 13,955)	21.05 (20.38, 21.73)	24.61 (23.89, 25.32)	54.34 (53.51, 55.17)	14.14 (13.56, 14.72)	57.11 (56.29, 57.93)	28.75 (28.00, 29.50)	51.62 (50.79, 52.45)	18.93 (18.28, 19.58)	29.45 (28.69, 30.20)
1940–1944 (*N* = 11,302)	22.85 (22.08, 23.63)	26.75 (25.93, 27.56)	50.40 (49.48, 51.32)	15.01 (14.35, 15.66)	59.61 (58.70, 60.51)	25.38 (24.58, 26.19)	56.60 (55.69, 57.51)	18.17 (17.46, 18.88)	25.23 (24.42, 26.03)
1945–1949 (*N* = 5,592)	24.20 (22.85, 25.08)	26.10 (24.94, 27.24)	49.70 (48.40, 51.02)	14.54 (13.61, 15.46)	60.68 (59.40, 61.96)	24.78 (23.65, 25.92)	59.71 (58.42, 60.99)	17.01 (16.02, 17.99)	23.28 (22.18, 24.39)
**Change across cohorts**									
Unadjusted RR	1.18*** (1.18, 1.20)	1.10** (1.09, 1.11)	0.84*** (0.83, 0.85)	1.04*** (1.03, 1.06)	1.13*** (1.11, 1.14)	0.86*** (0.85, 0.87)	1.25*** (1.23, 1.26)	0.94*** (0.93, 0.95)	0.81*** (0.80, 0.82)
Adjusted^α^ RR	1.21*** (1.20, 1.23)	1.10*** (1.09, 1.11)	0.83*** (0.82, 0.84)	1.09*** (1.07, 1.11)	1.09*** (1.08, 1.10)	0.86*** (0.85, 0.87)	1.24*** (1.23, 1.25)	0.95*** (0.94, 0.96)	0.82*** (0.81, 0.83)

***P < 0.0001, **P < 0.001. CI, confidence interval; RR, risk ratio.

^α^RR values are adjusted for gender, ethnicity, marital status, BMI, education, immigration status and household income. All percentages are weighted.

### Associations between lifestyle factors and depression across birth cohorts

The total weighted frequency of depression in all study participants was 2.35% (95% CI: 2.24–2.55). Table 3 shows the rates of depression by health risk behaviors across birth cohorts. The prevalence of depression in those born in the earlier cohort was 1.01 times that of those in more recent cohorts among active people (*P* < 0.05), and 1.17 times among inactive people (*P* < 0.0001). Similarly, the increase in depression rates was also reflected in other lifestyle factors (current smoker: RR = 1.21, *P* < 0.0001; non-smoker: RR = 1.10, *P* < 0.001; regular drinker: RR = 1.14, *P* < 0.0001; non-drinker: RR = 1.08, *P* < 0.001). The prevalence of depression among “former smokers” and “non-smokers” was lower than in “current smokers,” and the increased rate of depression in these two smoking groups was lower than in the “current smoker” group as we moved from earlier to more recent cohorts.

**TABLE 3:** Prevalence of depression by physical activity index, smoking status, and type of drinker across birth cohorts for Canadian residents 65+.

Birth cohort	Physical activity index			Smoking status			Type of drinker		
			
	Active rate, % (95% CI)	Moderate rate, % (95% CI)	Inactive rate, % (95% CI)	Current smoker rate, % (95% CI)	Former smoker rate, % (95% CI)	Non-smoker rate, % (95% CI)	Regular drinker rate, % (95% CI)	Occasional drinker rate, % (95% CI)	Non-drinker rate, % (95% CI)
1910–1914 (*N* = 542)	–	2.08 (1.24, 4.39)	2.75 (1.21, 4.28)	2.13 (0.15, 4.41)	2.28 (0.67, 3.88)	2.24 (0.59, 3.90)	1.79 (0.06, 2.90)	2.21 (0.63, 3.29)	1.65 (0.21, 3.08)
1915–1919 (*N* = 8,703)	1.45 (0.63, 2.26)	1.64 (0.98, 2.31)	2.19 (1.84, 2.55)	2.78 (1.58, 3.99)	2.10 (1.64, 2.56)	1.85 (1.44, 2.25)	1.65 (1.15, 2.15)	2.37 (1.70, 3.04)	2.10 (1.67, 2.53)
1920–1924 (*N* = 11,920)	1.61 (0.98, 2.23)	1.25 (0.79, 1.70)	2.64 (2.29, 3.00)	4.37 (3.26, 5.48)	2.17 (1.79, 2.55)	1.73 (1.37, 2.10)	1.88 (1.49, 2.29)	2.29 (1.72, 2.85)	2.52 (2.07, 2.96)
1925–1929 (*N* = 18,075)	1.32 (0.90, 1.73)	1.97 (1.53, 2.40)	2.87 (2.56, 3.17)	3.73 (2.93, 4.52)	2.24 (1.94, 2.54)	2.25 (1.89, 2.61)	2.21 (1.88, 2.54)	2.38 (1.90, 2.86)	2.71 (2.31, 3.10)
1930–1934 (*N* = 18,586)	1.80 (1.35, 2.24)	2.10 (1.68, 2.53)	3.08 (2.75, 3.40)	4.44 (3.62, 5.26)	2.38 (2.08, 2.68)	2.27 (1.90, 2.64)	2.26 (1.95, 2.58)	2.83 (2.31, 3.36)	2.98 (2.55, 3.40)
1935–1939 (*N* = 13,955)	1.30 (0.88, 1.70)	1.63 (1.21, 2.05)	3.28 (2.88, 3.68)	3.45 (2.64, 4.25)	2.33 (2.00, 2.67)	2.22 (1.76, 2.67)	2.07 (1.74, 2.40)	2.81 (2.17, 3.43)	2.92 (2.41, 3.44)
1940–1944 (*N* = 11,302)	2.09 (1.54, 2.64)	2.61 (2.04, 3.18)	3.44 (2.97, 3.91)	4.72 (3.71, 5.73)	2.58 (2.20, 2.96)	2.61 (2.03, 3.20)	2.38 (2.00, 2.75)	3.46 (2.67, 4.25)	3.72 (3.02, 4.41)
1945–1949 (*N* = 5,592)	2.37 (1.55, 3.18)	2.26 (1.50, 3.03)	4.78 (3.99, 5.58)	5.78 (4.17, 7.39)	3.45 (2.83, 4.06)	2.45 (1.64, 3.27)	3.23 (2.63, 3.83)	4.10 (2.84, 5.36)	3.92 (2.86, 4.97)
**Change across cohorts**									
Unadjusted RR	0.97 (0.89, 1.05)	1.05 (0.98, 1.13)	1.11*** (1.07, 1.15)	1.15*** (1.08, 1.23)	1.03 (0.99, 1.10)	1.05 (0.99, 1.11)	1.07** (1.02, 1.13)	1.13*** (1.07, 1.21)	1.07** (1.02, 1.12)
Adjusted^α^ RR	1.01* (1.00, 1.11)	1.12** (1.04, 1.21)	1.17*** (1.13, 1.22)	1.21*** (1.12, 1.30)	1.07** (1.03, 1.12)	1.10** (1.03, 1.16)	1.14*** (1.08, 1.20)	1.23*** (1.16, 1.32)	1.08** (1.02, 1.14)

***P < 0.0001, **P < 0.001, *P < 0.05. CI, confidence interval; PR, prevalence rate.

^α^RR values are adjusted for gender, marital status, education, immigration status, and household income.

**FIGURE 2:**
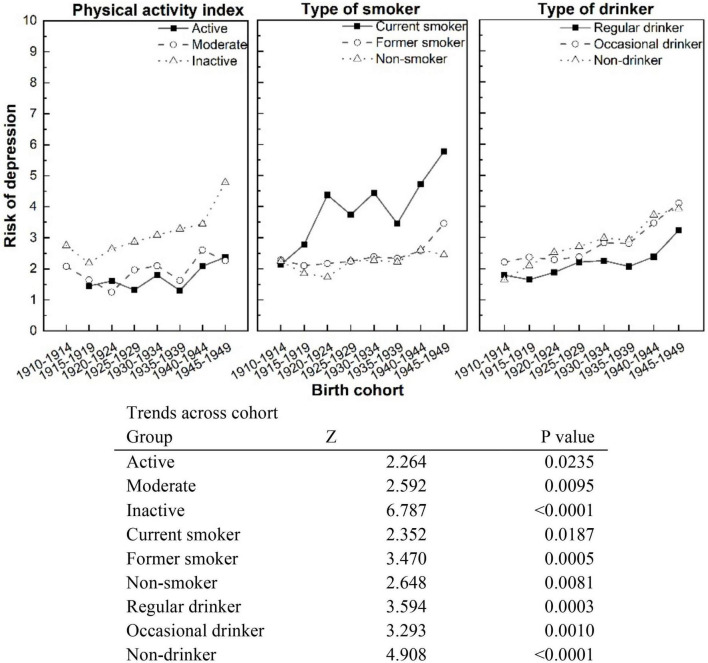
Trends in rates of depression by physical activity index, smoking status, and type of drinker across birth cohorts, Canadian residents 65+.

Figure 2 shows the trends in depression rate by health risk behaviors across eight birth cohorts. It indicated that depression was significantly increasing at the level of *P* < 0.05 across all birth cohorts, irrespective of health risk behavior status. The rate of depression among Canadian seniors peaked in the more recent 1940s birth cohorts.

Table 4 shows the associations between depression and health risk behaviors in the eight birth cohorts. The risk that “*physically inactive*” seniors would be depressed was 1.16–3.49 times that of “*active*” seniors in all the birth cohorts. The RR was highest in the 1925–1929 birth cohort (RR = 3.49, *P* < 0.0001). Likewise, the prevalence of depression among seniors who were “*current smokers*” was higher than among seniors who were “*non-smokers*” in all birth cohorts. These RRs between seniors who were “*current smokers*” and “*non-smokers*” ranged from 1.28 to 2.63 (all *P*-values < 0.05). Interestingly, for seniors born between 1915 and 1940, “*regular drinkers*” were associated with a 0.43- to 0.86-fold decrease in depression rate compared with those who were “*non-drinkers*.” But that was reversed for seniors born in 1945–1949; the depression rate was greater at 1.09 times for “*regular drinkers*” than for “*non-drinkers*” (*P* < 0.05).

**TABLE 4:** Unadjusted and adjusted relative risk of depression by physical activity index, smoking status, and type of drinker across eight birth cohorts for Canadian residents 65+.

Birth year	Physical activity index			Smoking status			Type of drinker		
			
	Active	Moderate	Inactive	Current smoker	Former smoker	Non-smoker	Regular drinker	Occasional drinker	Non-drinker
**1910–1914 (*N* = 542)**									
Unadjusted RR (95% CI)	–	–	–	1.66 (1.04, 11.15)	1.30 (0.37, 4.54)	1.00	1.27 (0.30, 5.33)	1.52 (0.33, 7.03)	1.00
Adjusted^α^ RR (95% CI)	–	–	–	1.38* (1.02, 8.73)	1.09 (0.24, 5.00)	1.00	1.34 (0.26, 6.78)	1.73 (0.31, 9.58)	1.00
**1915–1919 (*N* = 8,703)**									
Unadjusted RR (95% CI)	1.00	0.80 (0.41, 1.59)	0.87 (0.50, 1.51)	1.02 (0.53, 1.97)	1.24 (0.86, 1.80)	1.00	0.61* (0.40, 0.94)	0.85 (0.53, 1.32)	1.00
Adjusted^α^ RR (95% CI)	1.00	0.86 (0.46, 1.62)	1.16* (1.09, 1.95)	1.914* (1.484, 2.722)	1.12 (0.76, 1.63)	1.000	0.43*** (0.28, 0.69)	0.76 (0.49, 1.19)	1.00
**1920–1924 (*N* = 11,920)**									
Unadjusted RR (95% CI)	1.00	0.32*** (0.18, 0.58)	1.11 (0.70, 1.47)	2.13*** (1.48, 3.07)	0.95 (0.70, 1.30)	1.00	0.63** (0.46, 0.87)	0.83 (0.58, 1.18)	1.00
Adjusted^α^ RR (95% CI)	1.00	0.31*** (0.18, 0.56)	1.23* (1.04, 1.37)	2.25*** (1.55, 3.26)	1.10 (0.80, 1.53)	1.00	0.86** (0.61, 1.00)	0.88 (0.62, 1.25)	1.00
**1925–1929 (*N* = 18,075)**									
Unadjusted RR (95% CI)	1.00	0.96 (0.64, 1.43)	1.56** (1.12, 2.17)	1.32 (0.94, 1.87)	1.06 (0.81, 1.38)	1.00	0.69*** (0.53, 0.91)	1.17 (0.86, 1.58)	1.00
Adjusted^α^ RR (95% CI)	1.00	0.95 (0.64, 1.40)	1.46* (1.05, 2.03)	1.52* (1.08, 2.18)	1.42* (1.08, 1.87)	1.00	0.83* (0.63, 1.00)	1.23 (0.92, 1.65)	1.00
**1930–1934 (*N* = 18,586)**									
Unadjusted RR (95% CI)	1.00	2.71*** (1.56, 4.71)	3.76** (2.27, 6.24)	1.42* (1.03, 1.96)	0.76* (0.59, 0.98)	1.00	0.53*** (0.41, 0.69)	0.85 (0.63, 1.15)	1.00
Adjusted^α^ RR (95% CI)	1.00	2.56** (1.49, 4.40)	3.49* (2.12, 5.73)	1.54** (1.11, 2.14)	0.92 (0.71, 1.21)	1.00	0.56*** (0.43, 0.74)	0.81 (0.60, 1.09)	1.00
**1935–1939 (*N* = 13,955)**									
Unadjusted RR (95% CI)	1.00	2.39** (1.36, 4.18)	3.85*** (2.33, 6.38)	1.22 (0.82, 1.80)	0.76 (0.57, 1.20)	1.00	0.52*** (0.39, 0.70)	0.91 (0.64, 1.30)	1.00
Adjusted^α^ RR (95% CI)	1.00	2.18** (1.25, 3.80)	3.39*** (2.04, 5.61)	1.28* (1.06, 1.89)	0.96 (0.71, 1.29)	1.00	0.68* (0.50, 0.92)	0.91 (0.64, 1.29)	1.00
**1940–1944 (*N* = 11,302)**									
Unadjusted RR (95% CI)	1.00	1.32 (0.82, 2.15)	1.98** (1.30, 3.02)	2.20*** (1.39, 3.49)	1.32 (0.90, 1.93)	1.00	0.69* (0.49, 0.97)	1.36 (0.92, 2.01)	1.00
Adjusted^α^ RR (95% CI)	1.00	1.22 (0.77, 1.93)	1.87* (1.25, 2.80)	2.63*** (1.70, 4.07)	1.63** (1.14, 2.32)	1.00	0.80 (0.57, 1.12)	1.25 (0.86, 1.81)	1.00
**1945–1949 (*N* = 5,592)**									
Unadjusted RR (95% CI)	1.00	0.83 (0.40, 1.73)	2.62*** (1.51, 4.55)	3.01*** (1.69, 5.36)	1.17 (0.71, 1.95)	1.00	0.79 (0.48, 1.31)	1.48 (0.83, 2.64)	1.00
Adjusted^α^ RR (95% CI)	1.00	0.80 (0.39, 1.62)	2.31*** (1.34, 3.98)	2.97*** (1.65, 5.33)	1.37 (0.83, 2.28)	1.00	1.09* (1.01, 1.82)	1.57 (0.89, 2.76)	1.00

*P < 0.05, **P < 0.01, ***P < 0.0001. CI, confidence interval. RR, risk ratio.

^α^RR values are adjusted for gender, marital status, education, immigration status, and household income.

### The trend in the risk difference of depression in lifestyle factors and across cohorts

The risk difference of depression between relatively unhealthy risk behaviors groups–(moderately active and inactive, current and former smoker, regular and occasional drinker)–and healthy behaviors groups (active, non-smoker, and non-drinker) is shown in Table 5. These risk differences can be understood as the additional risks caused by unhealthy behavior in each birth cohort.

**TABLE 5:** Risk difference for depression by physical activity index, smoking status, and type of drinker across birth cohorts for Canadian residents 65+.

Birth year	Physical activity index		Smoking status		Type of drinker	
			
	Moderate	Inactive	Current smoker	Former smoker	Regular drinker	Occasional drinker
						
	Risk difference % (95% CI)	Risk difference % (95% CI)	Risk difference % (95% CI)	Risk difference % (95% CI)	Risk difference % (95% CI)	Risk difference % (95% CI)
1910–1914 (*N* = 542)	–	–	−0.85 (−1.89, 1.19)	−0.12 (−0.71, 0.48)	0.28 (−0.33, 0.89)	1.26*** (0.69, 1.83)
1915–1919 (*N* = 8,703)	0.18 (−0.16, 0.52)	0.53*** (0.24, 0.82)	0.35** (0.10, 0.59)	0.14 (−0.28, 0.30)	−0.28** (−0.46, −0.08)	0.12 (−0.05, 0.29)
1920–1924 (*N* = 11,920)	−0.28* (−0.54, −0.02)	0.44*** (0.24, 0.64)	1.03*** (0.87, 1.19)	0.41*** (0.28, 0.55)	−0.11 (−0.25, 0.03)	−0.04 (−0.19, 0.13)
1925–1929 (*N* = 18,075)	0.37*** (0.18, 0.57)	0.75*** (0.58, 0.92)	0.59*** (0.45, 0.73)	0.17** (0.06, 0.28)	−0.09 (−0.20, 0.02)	−0.12 (−0.25, 0.10)
1930–1934 (*N* = 18,586)	0.13 (−0.43, 0.31)	0.49*** (0.34, 0.64)	0.75*** (0.61, 0.88)	0.23*** (0.12, 0.35)	−0.15* (−0.26, −0.03)	−0.05 (−0.18, 0.78)
1935–1939 (*N* = 13,955)	0.19 (−0.28, 0.40)	0.87*** (0.69, 1.05)	0.49*** (0.32, 0.66)	0.24*** (0.11, 0.38)	−0.16* (−0.29, −0.03)	−0.07 (−0.22, 0.08)
1940–1944 (*N* = 11,302)	0.18 (−0.02, 0.39)	0.42*** (0.24, 0.59)	0.69*** (0.50, 0.88)	0.15 (−0.01, 0.31)	−0.24** (−0.39, −0.08)	−0.12 (−0.29, 0.06)
1945–1949 (*N* = 5,592)	−0.11 (−0.46, 0.23)	0.57*** (0.28, 0.85)	0.94*** (0.62, 1.25)	0.54*** (0.26, 0.81)	0.07 (−0.18, 0.32)	0.05 (−0.25, 0.34)
**Change across cohorts**						
Unadjusted RR	1.80 (0.88, 2.18)	1.80*** (1.49, 2.18)	1.63*** (1.37, 1.93)	1.01 (0.88, 1.15)	0.66*** (0.58, 0.76)	1.03 (0.88, 1.20)
Adjusted^α^ RR	1.06 (0.85, 1.33)	1.67*** (1.39, 2.01)	1.79*** (1.50, 2.12)	1.03 (0.97, 1.41)	0.78*** (0.68, 0.90)	1.06 (0.91, 1.23)

^α^RR values are adjusted for gender, marital status, education, immigration status and household income. *P < 0.05, **P < 0.01, ***P < 0.0001. CI, confidence interval; RR, risk ratio.

First, physical inactivity was associated with depression in each cohort, and associations systematically increased across all eight birth cohorts (RR = 1.67, *P* < 0.0001). Similarly, former smokers were indistinguishable from non-smokers regarding depression across birth cohorts (RR = 1.03, *P* > 0.05). But smoking-attributable depression risk for current smokers showed an increasing linear trend across cohorts (RR = 1.79, *P* < 0.0001). A decreasing association between birth cohort and risk difference in depression between regular drinkers and non-drinkers was observed (RR = 0.79, *P* < 0.0001).

### Associations between lifestyles and depression across survey years

We also analyzed the frequency of health behaviors and depression across survey years (Supplementary Tables 2–5). The frequency of “physically active” seniors showed an increasing linear trend from 1994 to 2014 (RR = 1.05, *P* < 0.0001). There has been a steady decrease in the proportion of “current smokers” (RR = 0.96, *P* < 0.0001). The prevalence of regular drinkers has been on the rise since the early 21st century and peaked in 2009 survey year (RR = 1.02, *P* < 0.0001) (Supplementary Table 2). The prevalence of depression declined in seniors who were physically active (RR = 0.97, *P* < 0.0001), former smokers (RR = 0.10, *P* < 0.05), and non-drinkers (RR = 0.10, *P* < 0.001) from 1994 to 2014 (Supplementary Table 3). Supplementary Table 4 shows that, among Canadian seniors, physical inactivity and smoking were progressively associated with depression since the early 2000s. For seniors who were regular drinkers, the risk of depression decreased by approximately 3% to 68% compared to non-drinkers (RR ranging from 0.32 to 0.97, all *P*-values < 0.05), partially no doubt reflecting the increased number of Canadian seniors who had become regular drinkers. According to the risk difference results, among seniors, both physical inactivity and smoking each contributed to depression (RR = 1.14, *P* < 0.001) and (RR = 1.12, *P* < 0.05), respectively, and that contribution increased as we moved through the survey years from 1994 to 2014 (Supplementary Table 5).

## Discussion

This study compared the relationship between health risk behaviors and depression among Canadians 65+ born at various times through the first part of the 20th century. By comparing these birth cohorts, we can seek the effects arising from the historical experience of various birth cohorts. Canadian 65+ born in more recent cohorts, in the 1940s, are more physically active but smoke and drink more than those born earlier in the century. The prevalence of depression increased in all health risk behavior groups from earlier to more recently born cohorts, specifically for current smokers and inactive people. We also found that inactivity/smoking-attributable depression showed an increasing linear trend from earlier to recent birth cohorts. Regular drinking was negatively associated with depression, except for those born in the 1940s. To our knowledge, the current study is the first to examine the broad link between health risk behaviors and depression among older adults across birth cohorts in large samples of the general population.

More and more older Canadians are adopting healthy lifestyles in the late 20th and early 21st centuries (39–41). In the current study, the overall drop in physical inactivity and cigarette consumption from 1994 to 2014 seemed likely to be associated with the strikingly greater public awareness of the health hazards of physical inactivity and smoking. A wealth of continuing public health warnings, education, and specific prohibitions on unhealthy lifestyles have been strongly supported by national and provincial governments. From the cohort analysis, it can still be observed that seniors born in the more recent cohort are more likely to smoke and be regular drinkers of alcohol. A diversity of studies has recognized the inverse relationship between smoking and age among older adults (42, 43). As the country’s population ages and we transition from birth cohorts with more permissive smoking norms to those with more restrictive norms, we expect that fewer seniors will be smoking cigarettes, but the role of smoking in depression will increase. However, currently, a greater proportion of Canadian seniors born in the 1940s continue to smoke cigarettes in spite of mounting societal pressure not to do so. There has been a substantial increase in the number of seniors who are now former smokers across birth cohorts; this is especially true for those seniors born in more recent cohorts. While quitting smoking is still advantageous for older people, prevention and early intervention are unquestionably more advantageous. Obviously, measures to improve wellbeing in older adults’ life should be implemented as early as possible. The results of this study indicate that alcohol consumption is still a major public health issue in Canada. As we can see, seniors born in the more recent birth cohorts consume alcohol more often than those born earlier in the 20th century. From a prevention perspective, more attention needs to be paid to Canada’s Low-Risk Alcohol Drinking Guidelines for Older Adults and the recommended alcohol consumption limits (44).

Of key public health interest is how social norms shape older individuals’ physical and mental health. Both cohort-specific norms and individuals’ attitudes or beliefs affect behavior factors and impact both physical and mental health. The “birth cohort effect” in our analysis reflects how these social, economic, and political resources to which people are exposed as they progress through their life journey significantly influence their health. They become essential exposures in the development of lifestyle behaviors and psychological outcomes. The more recently born seniors have a clinically higher risk for depression, leading to increased depression rates across all behavioral groups.

People born in more recent cohorts share the highest risk for inactivity-attributed risk for depression; this is especially true for seniors born in the 1930s. In Canada, people born in the 1930s are the parents of baby boomers (those born after World War II). They had larger-sized families, which contributed to the baby boom in Canada. They also experienced the Depression in the 1930s and World War II (1941–1945) in youth and as young adults. The antidepressant effects of exercise have been confirmed by extensive research (45–47). The benefits of physical activity may be more important for seniors because of the high risks of functional disability and physical and mental illnesses.

Our analysis of the change across survey years showed that cigarette smoking was consistently and progressively associated with depression among seniors from 1994 to 2014. Over time, as cigarette smoking became less prevalent among older adults, a stronger association with depression became evident. Such a model is consistent with the epidemiologic literature on tobacco consumption (48, 49). A historical trend analysis concluded that when cigarette consumption declined, its positive relationship with depression became more robust at the population level (50). The cohort results in this study also show that the relationship between smoking and depression increased among seniors from those born in the early 20th century to more recent birth cohorts born mid-century. The corresponding link between tobacco use and psychopathology has also been documented by a study on the trends of psychopathology among cocaine abusers (51). Some psychosocial and genetic factors can explain the increasing comorbidity with tobacco use and depression across birth cohorts. Research shows that members of more recent-born cohorts are prone to deviant behaviors and have more genetic liabilities to addiction than earlier-born cohorts (30, 52–54). It is also possible that the cigarette smoking-depression link is underestimated because of the possibility of the differential mortality effects of cigarette smoking. More specifically, older smokers with depression have higher mortality in earlier-birth cohorts than those in more recent-born cohorts. A prospective cohort study could be used to address the issue.

Previous studies on the link between light-to-moderate alcohol intake and psychological consequences in adults have produced inconsistent results. For example, a literature review suggested that moderate doses of alcohol might be beneficial to psychological wellbeing (55). Some studies might explain this as alcohol drinking resulting in stress-reduction effects that lower mental illness susceptibility. In contrast, García-Esquinas et al. (56) found no consistent protective effects of light to moderate alcohol consumption on depression in older adults. Future research should examine whether and how changes in alcohol consumption patterns affect depression risk by using repeated measures of alcohol intake and taking into account cultural norms regarding alcohol use and changes in those norms.

Cohort analysis reflects the unique social, economic, and political environments of generations born in different time periods but of the same chronological age. The period analysis reflects the general social environment of all individuals living at a specific time in history (57). Our study explored the changes in associations between health risk behaviors and depression among Canadian seniors born in different birth cohorts from the early to the mid-20th century. Our study is based on extremely large and representative Canadian seniors’ population samples. These findings can be generalized and help inform public health planning and interventions in Canada.

### Limitations

Despite being able to derive birth cohort analysis from a series of cross-sectional, nationally representative surveys, this study has several limitations. The first limitation is that the current study did not look at a multi-dimensional analysis from longitudinal survey data when drawing inferences about national trends, though little such national longitudinal data exists. We explored these changing associations based on annual cross-sectional observational studies, so our results could not infer causality. Secondly, although our sample comes from national health surveys, it is problematic whether our study results can be spread to other countries due to cultural, social, economic, and political environmental differences regarding the health risk behaviors studied here. Thirdly, changes due to aging itself have not been analyzed in the current study.

## Conclusion

The prevalence of depression increased among Canadian seniors from the earlier- to recent-born cohorts. The contribution of physical inactivity and cigarette smoking to depression prevalence is a greater risk for Canadian seniors born in more recently born cohorts as opposed to those born earlier in the 20th century. Smoking has become more firmly established as a risk factor for depression in the more recent birth cohorts. In contrast, moderate alcohol use was associated with a decreased risk of depression in earlier birth cohorts, but the strength of that connection has diminished, becoming non-existent in the most recent birth cohort. Identifying changing relationships between health risk behaviors and depression is meaningful for developing public health strategies and allocating health resources for older Canadians.

## Data availability statement

The original contributions presented in this study are included in the article/Supplementary material, further inquiries can be directed to the corresponding author.

## Ethics statement

In accordance with local legislation and institutional requirements ethical review and approval was not required for this study. The patients/participants provided their written informed consent to participate in the original sources surveys from which this study’s data is derived.

## Author contributions

Both authors contributed to the design of the study, contributed to further revisions of the manuscript, and approved the final submitted version of the manuscript. GY was responsible for the acquisition and analyses of the data and prepared the first draft of the manuscript. CD’A critically reviewed the initial draft of the manuscript.
