# Supplementary material for: The changing relationship between health risk behaviors and depression among birth cohorts of Canadians 65+, 1994–2014
Source: Front Psychiatry. 2022 Dec 21;13:1078161. doi: 10.3389/fpsyt.2022.1078161 (PMC9810750; doi:10.3389/fpsyt.2022.1078161)
Supplement: Supplementary file 1 [file Table_1.DOCX]

**Table S1.** Collection of survey data on Major Depression using the WHO-CIDI-SF instrument in the Provinces and Territories of Canada, by Survey/year, 1994-2014

| Survey, Year | BC | AB | SK | MB | ONT | QUE | NB | NS | PEI | NLFD | YT | NT | NU* | Sample Size |
| --- | --- | --- | --- | --- | --- | --- | --- | --- | --- | --- | --- | --- | --- | --- |
| NPHS, 1994/1995 | √ | √ | √ | √ | √ | √ | √ | √ | √ | √ | × | × | × | 2,792 |
| NPHS, 1996/1997 | √ | √ | √ | √ | √ | √ | √ | √ | √ | √ | × | × | × | 8,877 |
| NPHS, 1998/1999 | √ | √ | √ | √ | √ | √ | √ | √ | √ | √ | × | × | × | 2,436 |
| CCHS, 2001 | √ | √ | √ | √ | √ | √ | √ | √ | √ | √ | √ | √ | √ | 18,358 |
| CCHS, 2003 | × | √ | × | × | √ | × | √ | × | √ | √ | √ | √ | √ | 7,259 |
| CCHS, 2005 | √ | √ | √ | × | × | √ | × | √ | √ | × | × | × | × | 10,817 |
| CCHS, 2007/2008 | × | √ | × | × | × | √ | √ | √ | × | × | × | × | × | 7,331 |
| CCHS, 2009/2010 | √ | √ | √ | × | × | √ | × | × | √ | × | × | × | × | 9,959 |
| CCHS, 2011/2012 | × | × | √ | × | × | × | √ | √ | √ | √ | × | × | × | 5,415 |
| CCHS, 2013/2014 | × | × | × | √ | × | √ | × | √ | √ | √ | × | × | × | 10,455 |
| CCHS, 2014 | × | × | × | √ | × | √ | × | √ | √ | √ | × | × | × | 5,406 |

Abbreviations – Province: BC = British Columbia, AB = Alberta, SK = Saskatchewan, MB = Manitoba, ONT = Ontario, Que = Quebec, NB = New Brunswick, NS = Nova Scotia, PEI = Prince Edward Island, NLFD = Newfoundland and Labrador, YT = Yukon Territory, NT = Northwest.

Footnote* Nunavut became a separate territory of Canada in 1999.
