# Supplementary material for: The changing relationship between health risk behaviors and depression among birth cohorts of Canadians 65+, 1994–2014
Source: Front Psychiatry. 2022 Dec 21;13:1078161. doi: 10.3389/fpsyt.2022.1078161 (PMC9810750; doi:10.3389/fpsyt.2022.1078161)
Supplement: Supplementary file 2 [file Table_2.DOCX]

**Table S2**. Frequency of physical activity index, smoking status, and type of drinkers across survey year, Canadian residents 65+

| Survey year | Physical activity index | | |  | Smoking status | | |  | Type of drinker | | |
| --- | --- | --- | --- | --- | --- | --- | --- | --- | --- | --- | --- |
|  | Active% (*95% CI*) | Moderate% (*95% CI*) | Inactive% (*95% CI*) |  | Current smoker% (*95% CI*) | Former smoker% (*95% CI*) | Non-smoker% (*95% CI*) |  | Regular drinker% (*95% CI*) | Occasional drinker% (*95% CI*) | Non-drinker% (*95% CI*) |
| 1994  (N=2792) | 13.83 | 18.12 | 68.05 |  | 15.76 | 42.48 | 41.76 |  | 36.14 | 21.06 | 42.80 |
| 1996  (N=8877) | 14.00 | 20.28 | 65.72 |  | 14.86 | 39.72 | 45.42 |  | 37.34 | 22.43 | 40.23 |
| 1998  (N=2436) | 15.02 | 21.22 | 63.75 |  | 14.24 | 47.33 | 38.42 |  | 35.76 | 22.50 | 41.75 |
| 2001  (N=18358) | 16.53 | 21.21 | 62.27 |  | 13.30 | 51.88 | 34.81 |  | 41.26 | 22.32 | 36.42 |
| 2003  (N=7259) | 18.20 | 22.18 | 59.62 |  | 11.99 | 53.71 | 34.30 |  | 45.17 | 19.86 | 34.96 |
| 2005  (N=10817) | 18.77 | 24.00 | 57.23 |  | 12.31 | 55.37 | 32.32 |  | 46.60 | 20.82 | 32.57 |
| 2007  (N=7331) | 18.29 | 22.45 | 59.26 |  | 12.26 | 56.43 | 31.31 |  | 48.63 | 19.72 | 31.65 |
| 2009  (N=9959) | 20.11 | 25.13 | 54.75 |  | 12.39 | 56.50 | 31.11 |  | 53.25 | 18.76 | 27.99 |
| 2011  (N=5415) | 16.37 | 22.23 | 61.40 |  | 11.82 | 56.73 | 31.45 |  | 42.21 | 20.24 | 37.55 |
| 2013  (N=10455) | 19.07 | 24.36 | 56.57 |  | 10.85 | 59.33 | 29.82 |  | 51.86 | 18.38 | 29.76 |
| 2014  (N=5406) | 19.37 | 24.29 | 56.34 |  | 10.75 | 59.77 | 29.49 |  | 52.33 | 18.44 | 29.23 |
| Change across years |  |  |  |  |  |  |  |  |  |  |  |
| Unadjusted RR | 1.031^***^  (1.026,1.036) | 1.031^**^  (1.026,1.036) | 0.982^***^  (0.980,0.984) |  | 0.967^***^  (0.961,0.973) | 1.033^***^  (1.031,1.035) | 0.958^***^  (0.955,0.962) |  | 1.038^***^  (1.035,1.040) | 0.977^***^  (0.972,0.981) | 0.963^***^  (0.960,0.966) |
| Adjusted^α^ RR | 1.046^***^  (1.041,1.052) | 1.023^**^  (1.019,1.028) | 0.982^***^  (0.979,0.986) |  | 0.958^***^  (0.951,0.964) | 1.023^***^  (1.021,1.025) | 0.964^***^  (0.961,0.968) |  | 1.023^***^  (1.020,1.025) | 0.972^***^ (0.967,0.977) | 0.952^***^ (0.949,0.955) |

***P<0.0001, **P<0.001

Abbreviation: CI, confidence interval. PR, prevalence rate

^α^ PR values are adjusted for gender, ethnicity, marital status, BMI, education, immigration status, and household income. All rates are weighted
