# Supplementary material for: The changing relationship between health risk behaviors and depression among birth cohorts of Canadians 65+, 1994–2014
Source: Front Psychiatry. 2022 Dec 21;13:1078161. doi: 10.3389/fpsyt.2022.1078161 (PMC9810750; doi:10.3389/fpsyt.2022.1078161)
Supplement: Supplementary file 3 [file Table_3.DOCX]

**Table S3.** Prevalence of depression by physical activity index, smoking status, and type of drinker across survey years, Canadian residents 65+

| Survey year | Physical activity index | | |  | Smoking status | | |  | Type of drinker | | |
| --- | --- | --- | --- | --- | --- | --- | --- | --- | --- | --- | --- |
|  | Active  Rate, %  (*95% CI*) | Moderate  Rate, %  (*95% CI*) | Inactive  Rate, %  (*95% CI*) |  | Current smoker  Rate, %  (*95% CI*) | Former smoker  Rate, %  (*95% CI*) | Non-smoker  Rate, %  (*95% CI*) |  | Regular drinker  Rate, %  (*95% CI*) | Occasional drinker  Rate, %  (*95% CI*) | Non-drinker  Rate, %  (*95% CI*) |
| 1994  (N=2792) | 1.81 | 3.16 | 3.38 |  | 4.77 | 3.54 | 2.06 |  | 2.97 | 3.40 | 3.10 |
| 1996  (N=8877) | 1.21 | 1.11 | 1.95 |  | 2.20 | 1.73 | 1.46 |  | 1.63 | 2.11 | 1.48 |
| 1998  (N=2436) | 1.37 | 0.58 | 2.96 |  | 4.03 | 1.91 | 1.92 |  | 1.15 | 2.74 | 2.85 |
| 2001  (N=18358) | 2.34 | 2.77 | 4.00 |  | 6.18 | 3.09 | 2.97 |  | 2.96 | 3.59 | 3.95 |
| 2003  (N=7259) | 1.21 | 1.68 | 2.20 |  | 3.33 | 1.64 | 1.81 |  | 1.65 | 2.08 | 2.13 |
| 2005  (N=10817) | 1.23 | 1.81 | 2.81 |  | 3.15 | 2.25 | 1.97 |  | 2.00 | 2.40 | 2.58 |
| 2007  (N=7331) | 1.19 | 1.88 | 2.74 |  | 3.56 | 2.01 | 2.22 |  | 1.82 | 2.28 | 2.93 |
| 2009  (N=9959) | 1.80 | 1.68 | 2.81 |  | 3.81 | 2.13 | 2.07 |  | 2.23 | 2.84 | 2.15 |
| 2011  (N=5415) | 1.10 | 1.90 | 3.43 |  | 5.26 | 2.65 | 1.85 |  | 2.42 | 2.38 | 3.21 |
| 2013  (N=10455) | 1.91 | 2.20 | 3.04 |  | 4.06 | 2.61 | 2.12 |  | 2.40 | 2.76 | 2.93 |
| 2014  (N=5406) | 2.10 | 1.60 | 3.05 |  | 3.61 | 2.48 | 2.20 |  | 2.33 | 2.81 | 2.66 |
| Change across years |  |  |  |  |  |  |  |  |  |  |  |
| Unadjusted RR | 0.961^***^  (0.958,0.963) | 1.002^*^  (1.000,1.004) | 1.028^***^  (1.022,1.036) |  | 1.051^***^  (1.048,1.053) | 0.996^***^  (0.994,0.998) | 1.016^***^  (1.014,1.017) |  | 1.029^***^  (1.028,1.031) | 1.030^***^  (1.028,1.032) | 0.997^***^  (0.995,0.998) |
| Adjusted^α^  RR | 0.968^***^  (0.965,0.971) | 1.019^***^  (1.017,1.022) | 1.037^***^  (1.036,1.038) |  | 1.064^***^  (1.061,1.066) | 0.998^*^  (0.997,0.999) | 1.028^***^  (1.026,1.029) |  | 1.041^***^  (1.040,1.043) | 1.063^***^ (1.061,1.065) | 0.988^***^ (0.986,0.989) |

*P<0.0001, **P<0.01

Abbreviation: CI, confidence interval. PR, prevalence rate

^α^ PR values are adjusted for gender, marital status, education, immigration status, language speaking and household income.
