# Supplementary material for: The changing relationship between health risk behaviors and depression among birth cohorts of Canadians 65+, 1994–2014
Source: Front Psychiatry. 2022 Dec 21;13:1078161. doi: 10.3389/fpsyt.2022.1078161 (PMC9810750; doi:10.3389/fpsyt.2022.1078161)
Supplement: Supplementary file 4 [file Table_4.DOCX]

**Table S4**. Unadjusted and adjusted Relative Risk of Depression by physical activity index, smoking status, type of drinker in survey years, Canadian residents 65+

| Survey year | Physical activity index | | | |  | | Smoking status | | |  | Type of drinker | | | | |
| --- | --- | --- | --- | --- | --- | --- | --- | --- | --- | --- | --- | --- | --- | --- | --- |
|  | Active | Moderate | Inactive |  | | Current smoker | | Former smoker | Non-smoker | | |  | Regular drinker | Occasional drinker | Non-drinker |
| 1994 (N=2792) |  |  |  |  | |  | |  |  | | |  |  |  |  |
| Unadjusted RR  (*95% CI*) | 1.000 | 1.347^***^  (1.307,1.877) | 1.829^***^  (1.782,1.389) |  | | 1.648^***^  (1.612,1.685) | | 1.663^***^  (1.634,1.692) | 1.000 | | |  | 0.689^***^  (0.677,0.701) | 1.062^***^  (1.043,1.082) | 1.000 |
| Adjusted ^α^ RR  (*95% CI*) | 1.000 | 1.139^***^  (1.104,1.174) | 1.437^***^  (1.399,1.475) |  | | 1.879^***^  (1.837,1.922) | | 2.263^***^  (2.223,2.304) | 1.000 | | |  | 0.938^***^  (0.921,0.955) | 1.162^***^  (1.141,1.141) | 1.000 |
| 1996 (N=8877) |  |  |  |  | |  | |  |  | | |  |  |  |  |
| Unadjusted RR  (*95% CI*) | 1.000 | 1.570^***^  (1.554,1.587) | 1.506^***^  (1.494,1.518) |  | | 1.273^***^  (1.243,1.311) | | 0.949  (0.929,0.970) | 1.000 | | |  | 0.704^***^  (0.687,0.721) | 1.666^***^  (1.628,1.704) | 1.000 |
| Adjusted ^α^ RR  (*95% CI*) | 1.000 | 1.580^***^  (1.563,1.597) | 1.582^***^  (1.569,1.596) |  | | 1.067^***^  (1.038,1.097) | | 0.763^***^  (0.745,0.782) | 1.000 | | |  | 0.612^***^  (0.596,0.628) | 1.668^***^  (1.630,1.707) | 1.000 |
| 1998 (N=2436) |  |  |  |  | |  | |  |  | | |  |  |  |  |
| Unadjusted RR  (*95% CI)* | 1.000 | 0.731  (0.709,0.755) | 1.860^***^  (1.815,1.960) |  | | 1.314^***^  (1.287,1.342) | | 0.848^**^  (0.834,0.862) | 1.000 | | |  | 0.340^***^  (0.333,0.346) | 0.734^***^  (0.721,0.747) | 1.000 |
| Adjusted ^α^ RR  (*95% CI*) | 1.000 | 0.753  (0.729,0.777) | 2.082^***^  (2.032,2.134) |  | | 1.487^***^  (1.456,1.519) | | 0.928^***^  (0.912,0.945) | 1.000 | | |  | 0.317^***^  (0.311,0.324) | 0.705^***^  (0.693,0.718) | 1.000 |
| 2001 (N=18358) |  |  |  |  | |  | |  |  | | |  |  |  |  |
| Unadjusted RR  (*95% CI*) | 1.000 | 1.306^***^  (0.273,1.340) | 2.039^***^  (1.996,2.084) |  | | 1.696^***^  (1.667,1.762) | | 0.796^***^  (0.785,0.808) | 1.000 | | |  | 0.752^***^  (0.741,0.763) | 0.873^***^  (0.858,0.888) | 1.000 |
| Adjusted ^α^ RR  (*95% CI*) | 1.000 | 1.266^***^  (1.234,1.299) | 1.782^***^  (1.743,1.821) |  | | 1.968^***^  (1.933,2.002) | | 1.017^*^  (1.001,1.032) | 1.000 | | |  | 0.965^***^  (0.950,0.980) | 0.922^***^  (0.906,0.938) | 1.000 |
| 2003 (N=7259) |  |  |  |  | |  | |  |  | | |  |  |  |  |
| Unadjusted RR  (*95% CI*) | 1.000 | 1.542^***^  (1.458,1.632) | 2.319^***^  (2.209,2.435) |  | | 1.647^***^  (1.579,1.719) | | 0.842^***^  (0.816,0.870) | 1.000 | | |  | 0.477^***^  (0.462,0.492) | 0.619^***^  (0.593,0.645) | 1.000 |
| Adjusted ^α^ RR  (*95% CI*) | 1.000 | 1.516^***^  (1.433,1.605) | 2.031^***^  (1.933,2.133) |  | | 1.715^***^  (1.641,1.792) | | 1.108^***^  (1.071,1.147) | 1.000 | | |  | 0.644^***^  (0.622,0.666) | 0.693^***^  (0.665,0.723) | 1.000 |
| 2005 (N=10817) |  |  |  |  | |  | |  |  | | |  |  |  |  |
| Unadjusted RR  (*95% CI*) | 1.000 | 1.295^***^  (1.235,1.358) | 2.447^***^  (2.351,2.546) |  | | 1.487^***^  (1.433,1.543) | | 0.988  (0.961,1.015) | 1.000 | | |  | 0.635^***^  (0.618,0.653) | 0.867^***^  (0.839,0.896) | 1.000 |
| Adjusted ^α^ RR  (*95% CI*) | 1.000 | 1.230^***^  (1.173,1.290) | 2.217^***^  (2.129,2.309) |  | | 1.655^***^  (1.594,1.718) | | 1.203^***^  (1.169,1.238) | 1.000 | | |  | 0.768^***^  (0.746,0.790) | 0.896^***^  (0.867,0.926) | 1.000 |
| 2007 (N=7331) |  |  |  |  | |  | |  |  | | |  |  |  |  |
| Unadjusted RR  (*95% CI*) | 1.000 | 2.114^***^  (2.001,2.233) | 3.376^***^  (3.216,3.544) |  | | 1.777^***^  (1.715,1.842) | | 0.933^***^  (0.907,0.961) | 1.000 | | |  | 0.723^***^  (0.702,0.743) | 0.897^***^  (0.865,0.929) | 1.000 |
| Adjusted ^α^ RR  (*95% CI*) | 1.000 | 1.988^***^  (1.881,2.100) | 3.120^***^  (2.971,3.276) |  | | 2.019^***^  (1.947,2.094) | | 1.222^***^  (1.186,1.259) | 1.000 | | |  | 0.911^***^  (0.884,0.938) | 0.899^***^  (0.867,0.931) | 1.000 |
| 2009 (N=9959) |  |  |  |  | |  | |  |  | | |  |  |  |  |
| Unadjusted RR  (*95% CI*) | 1.000 | 1.260^***^  (1.214,1.307) | 1.855^***^  (1.798,1.915) |  | | 1.684^***^  (1.630,1.741) | | 1.098^***^  (1.071,1.125) | 1.000 | | |  | 1.097^***^  (1.026,1.138) | 1.055^***^  (1.021,1.090) | 1.000 |
| Adjusted ^α^ RR  (*95% CI*) | 1.000 | 1.258^***^  (1.213,1.305) | 1.899^***^  (1.839,1.960) |  | | 2.290^***^  (2.214,2.369) | | 1.429^***^  (1.393,1.466) | 1.000 | | |  | 0.908^***^  (0.598,0.998) | 1.084^***^  (1.049,1.120) | 1.000 |
| 2011 (N=5415) |  |  |  |  | |  | |  |  | | |  |  |  |  |
| Unadjusted RR  (*95% CI*) | 1.000 | 1.250^***^  (1.173,1.331) | 1.558^***^  (1.476,1.544) |  | | 3.204^***^  (3.038,3.380) | | 1.419^***^  (1.356,1.486) | 1.000 | | |  | 0.450^***^  (0.432,0.469) | 0.842^***^  (0.805,0.881) | 1.000 |
| Adjusted ^α^ RR  (*95% CI*) | 1.000 | 1.242^***^  (1.166,1.323) | 1.340^***^  (1.268,1.416) |  | | 3.503^***^  (3.323,3.694) | | 1.617^***^  (1.543,1.694) | 1.000 | | |  | 0.548^***^  (0.525,0.573) | 0.852^***^  (0.815,0.891) | 1.000 |
| 2013 (N=10455) |  |  |  |  | |  | |  |  | | |  |  |  |  |
| Unadjusted RR  (*95% CI*) | 1.000 | 1.397^***^  (1.347,1.450) | 2.180^***^  (2.112,2.250) |  | | 1.967^***^  (1.910,2.026) | | 0.909^***^  (0.888,0.931) | 1.000 | | |  | 1.222^**^  (1.123,1.255) | 1.598^***^  (1.553,1.645) | 1.000 |
| Adjusted ^α^ RR  (*95% CI*) | 1.000 | 1.257^***^  (1.211,1.304) | 1.842^***^  (1.784,1.903) |  | | 2.243^***^  (2.176,2.311) | | 1.064^***^  (1.039,1.091) | 1.000 | | |  | 0.959^***^  (0.935,0.984) | 1.603^***^  (1.557,1.650) | 1.000 |
| 2014 (N=5406) |  |  |  |  | |  | |  |  | | |  |  |  |  |
| Unadjusted RR  (*95% CI*) | 1.000 | 1.037^***^  (1.001,1.078) | 2.443^***^  (2.369,2.519) |  | | 1.881^***^  (1.824,1.939) | | 1.017  (0.994,1.041) | 1.000 | | |  | 0.837***  (0.817,0.859) | 1.770***  (1.723,1.819) | 1.000 |
| Adjusted ^α^ RR  (*95% CI*) | 1.000 | 0.923^***^  (0.888,0.959) | 2.192^***^  (2.124,2.262) |  | | 2.156^***^  (2.089,2.224) | | 1.237^***^  (1.208,1.266) | 1.000 | | |  | 0.970^*^  (0.945,0.997) | 1.701^***^  (1.655,1.748) | 1.000 |

*P<0.05, **P<0.01, ***P<0.0001

Abbreviation: CI, confidence interval. RR, risk ratio

^α^ RR values are adjusted for gender, marital status, education, immigration status, language speaking and household income.
