# Supplementary material for: The changing relationship between health risk behaviors and depression among birth cohorts of Canadians 65+, 1994–2014
Source: Front Psychiatry. 2022 Dec 21;13:1078161. doi: 10.3389/fpsyt.2022.1078161 (PMC9810750; doi:10.3389/fpsyt.2022.1078161)
Supplement: Supplementary file 5 [file Table_5.DOCX]

**Table S5**. Risk difference of depression by physical activity index, smoking status, and type of drinker across survey years for Canadian residents 65+

| Birth year | Physical activity index | | | |  | | Smoking status | | | | | |  | | Type of drinker | | |  |
| --- | --- | --- | --- | --- | --- | --- | --- | --- | --- | --- | --- | --- | --- | --- | --- | --- | --- | --- |
|  | Moderate | | Inactive | |  | | Current smoker | | Former smoker | |  | | Regular drinker | | | Occasional drinker | | |
|  | *vs.* Active  % (*95% CI*) | | *vs.* Active  % (*95% CI*) | |  | | *vs*. non-smoker  % (*95% CI*) | | *vs.* non-smoker  % (*95% CI*) | |  | | *vs*. non-drinker  % (*95% CI*) | | | *vs.* non-drinker  % (*95% CI*) | | |
| 1994 (N=2792) | 0.30^***^  (0.27,0.33) | | 0.60^***^  (0.58,0.63) | |  | | 0.50^***^  (0.48,0.52) | | 0.51^***^  (0.49,0.53) | |  | | -0.37^***^  (-0.39,-0.36) | | | 0.06^***^  (0.04,0.08) | | |
| 1996 (N=8877) | -0.56^***^  (-0.59,-0.53) | | -0.68^***^  (-0.71,-0.66) | |  | | 0.24^***^  (0.21,0.27) | | -0.05^***^  (-0.07,-0.03) | |  | | -0.35^***^  (-0.37,-0.33) | | | 0.51^***^  (0.49,0.53) | | |
| 1998 (N=2436) | -0.31^***^  (-0.34,-0.28) | | 0.62^***^  (0.59,0.65) | |  | | 0.27^***^  (0.25,0.29) | | -0.17^***^  (-0.18,-0.15) | |  | | -1.08^***^  (-1.10,-1.06) | | | -0.31^***^  (-0.33,-0.29) | | |
| 2001 (N=18358) | 0.27^***^  (0.24,0.29) | | 0.71^***^  (0.69,0.73) | |  | | 0.53^***^  (0.51,0.55) | | -0.23^***^  (-0.24,-0.21) | |  | | -0.29^***^  (-0.30,-0.27) | | | -0.14^***^  (-0.15,-0.12) | | |
| 2003 (N=7259) | 0.43^***^  (0.37,0.49) | | 0.84^***^  (0.79,0.89) | |  | | 0.50^***^  (0.46,0.54) | | -0.17^***^  (-0.20,-0.15) | |  | | -0.74^***^  (-0.77,-0.71) | | | -0.48^***^  (-0.52,-0.44) | | |
| 2005 (N=10817) | 0.26^***^  (0.21,0.31) | | 0.89^***^  (0.86,0.94) | |  | | 0.40^***^  (0.36,0.43) | | -0.01  (-0.04,0.02) | |  | | -0.46^***^  (-0.48,-0.43) | | | -0.14^***^  (-0.18,-0.11) | | |
| 2007 (N=7331) | 0.75^***^  (0.69,0.80) | | 1.22^**^  (1.17,1.27) | |  | | 0.58^***^  (0.54,0.62) | | -0.07^***^  (-0.10,-0.04) | |  | | -0.33^***^  (-0.35,-0.30) | | | -0.11^***^  (-0.15,-0.07) | | |
| 2009 (N=9959) | 0.23^***^  (0.19,0.27) | | 0.61^***^  (0.57,0.65) | |  | | 0.52^***^  (0.49,0.55) | | 0.09^***^  (0.07,0.12) | |  | | -0.09^***^  (-0.11,-0.06) | | | 0.05^**^  (0.02,0.09) | | |
| 2011 (N=5415) | 0.23^***^  (0.16,0.29) | | 0.44^***^  (0.39,0.50) | |  | | 1.17^***^  (1.11,1.12) | | 0.35^***^  (0.30,0.39) | |  | | -0.80^***^  (-0.84,-0.76) | | | -0.17^***^  (-0.22,-0.13) | | |
| 2013 (N=10455) | 0.34^***^  (0.30,0.37) | | 0.80^***^  (0.75,0.81) | |  | | 0.68^***^  (0.64,0.71) | | -0.10^***^  (-0.20,-0.07) | |  | | -0.04^***^  (-0.07,-0.02) | | | 0.47^***^  (0.44,0.50) | | |
| 2014 (N=5406) | 0.04  (-0.02,0.08) | | 0.89^***^  (0.86,0.92) | |  | | 0.63^***^  (0.60,0.66) | | 0.02  (-0.01,0.04) | |  | | -0.18^***^  (-0.20,-0.16) | | | 0.57^***^  (0.54,0.60) | | |
| Change across years | |  | |  | |  | |  | |  | |  | |  | | |  |  |
| Unadjusted RR | | 0.890^***^  (0.872,0.908) | | 1.263^**^  (1.242,1.284) | |  | | 1.034^***^  (1.032,1.037) | | 0.980^***^  (0.978,0.982) | |  | | 1.032^***^  (1.030,1.035) | | | 1.034^***^  (1.031,1.039) |  |
| Adjusted ^α^ RR | | 1.040^***^  (1.037,1.044) | | 1.068^***^  (1.065,1.071) | |  | | 1.031^***^  (1.028,1.034) | | 0.981^***^  (0.978,0.984) | |  | | 1.036^***^  (1.034,1.038) | | | 1.028  (1.026,1.031) |  |

*P<0.05, **P<0.01, ***P<0.0001

Abbreviation: CI, confidence interval. PR, prevalence rate

^α^ Risk difference values are adjusted for gender, marital status, education, immigration status, and household income.
